# The association between systemic inflammation markers and paroxysmal atrial fibrillation

**DOI:** 10.1186/s12872-024-04004-9

**Published:** 2024-07-04

**Authors:** Xuechen Zhao, Lei Huang, Jianan Hu, Nake Jin, Jun Hong, Xudong Chen

**Affiliations:** Department of Cardiology, Ningbo Hangzhou Bay Hospital, 1155 Binhai 2nd Road, Hangzhou Bay New Area, Ningbo, 315336 China

**Keywords:** Systemic inflammation markers, Systemic immune inflammation index, System inflammation response index, Aggregate index of systemic inflammation, Paroxysmal atrial fibrillation

## Abstract

**Background:**

Systemic inflammation markers have recently been identified as being associated with cardiac disorders. However, limited research has been conducted to estimate the pre-diagnostic associations between these markers and paroxysmal atrial fibrillation (PAF). Our aim is to identify potential biomarkers for early detection of PAF.

**Methods:**

91 participants in the PAF group and 97 participants in the non-PAF group were included in this study. We investigated the correlations between three systemic inflammation markers, namely the systemic immune inflammation index (SII), system inflammation response index (SIRI), and aggregate index of systemic inflammation (AISI), and PAF.

**Results:**

The proportion of patients with PAF gradually increased with increasing logSII, logSIRI, and logAISI tertiles. Compared to those in the lowest tertiles, the PAF risks in the highest logSII and logSIRI tertiles were 3.2-fold and 2.9-fold, respectively. Conversely, there was no significant correlation observed between logAISI and PAF risk within the highest tertile of logAISI. The restricted cubic splines (RCS) analysis revealed a non-linear relationship between the elevation of systemic inflammation markers and PAF risk. Specifically, the incidence of PAF is respectively increased by 56%, 95%, and 150% for each standard deviation increase in these variables. The ROC curve analysis of logSII, logSIRI and logAISI showed that they had AUC of 0.6, 0.7 and 0.6, respectively. It also demonstrated favorable sensitivity and specificity of these systemic inflammation markers in detecting the presence of PAF.

**Conclusions:**

In conclusion, our study reveals significant positive correlations between SII, SIRI, and AISI with the incidence of PAF.

**Supplementary Information:**

The online version contains supplementary material available at 10.1186/s12872-024-04004-9.

## Introduction

Atrial fibrillation (AF) is the most prevalent cardiac arrhythmia, and its association with stroke, myocardial infarction, and heart failure leads to increased cardiovascular morbidity and mortality [1, 2]. The estimated prevalence of atrial fibrillation (AF) among adults ranges from 2 to 4%, with projections indicating an anticipated increase attributed to the extension of life expectancy [3]. However, the reported prevalence of previously undiagnosed AF among individuals aged over 60 is 20.1%, while a significant proportion (23.5%) of those who have received a diagnosis do not receive treatment with oral anticoagulants [4]. Paroxysmal atrial fibrillation (PAF) is estimated to occur in 25–60% of cases involving AF and is believed to precede the development of sustained AF. This progression can result in gradual changes to the electrical and structural properties of the atria, commonly referred to as “AF begets AF“ [5, 6]. Cryptogenic strokes account for 15–40% of ischemic strokes, and PAF is increasingly recognized as a potential etiology [7]. Therefore, the timely identification and diagnosis of PAF are imperative for the prevention of stroke.

In recent years, there has been a significant focus on the development and investigation of biomarkers with the capability to predict PAF. It has been demonstrated that there is an association between inflammatory activity and AF as well as its complications [8, 9]. The association between AF burden and inadequate maintenance of sinus rhythm has been evidenced through the examination of specific inflammatory indicators, including CRP and IL-6 [10]. The findings of extensive research indicate that both human and animal studies offer compelling evidence supporting the hypothesis that autoimmunity and inflammation may contribute to the pathogenesis of AF [8]. The presence of white blood cells, including their various subpopulations, along with platelets, is essential in the context of systemic inflammation. Recently, there have been studies that have brought attention to various indicators of inflammation throughout the body’s peripheral blood cells. These markers include the systemic immune inflammation index (SII), system inflammation response index (SIRI), and aggregate index of systemic inflammation (AISI). It is worth noting that these markers are linked to both cardiovascular and non-cardiovascular diseases [11–14].

Despite the existing evidence linking SII and SIRI to AF in stroke patients [12], there is a lack of research investigating the relationship between SII, SIRI, AISI, and PAF. Additionally, no studies have concurrently compared these three markers of systemic inflammation. Therefore, we conducted a retrospective analysis to gain deeper insights into the associations between SII, SIRI and AISI with PAF. The primary objective of this study is to accumulate evidence for potential biomarkers that can aid in the early detection of PAF.

## Materials and methods

### Study population

In this study, we consecutively enrolled patients with PAF, who were seen in the Department of Cardiology at our hospital between January 2020 and December 2022. The diagnosis of arrhythmia necessitated the inclusion of official medical records, a 12-lead ECG, or a 24-hour Holter recording, with its classification being based on internationally recognized consensus statements [15]. Simultaneously, we recruited hospitalized individuals with sinus rhythm (SR) as a control group. The eligible subjects for our study were provided with detailed information during outpatient follow-up and telephone contact to ensure their informed consent, and the study was conducted only after obtaining consent from the enrolled subjects. All participants provided informed consent by signing both paper forms (for outpatient follow-up) and electronic forms (for telephone follow-up).The study enrolled individuals aged over 18 years who were clinically diagnosed with PAF and provided informed consent to participate in the study by submitting a signed informed consent form. Exclusion criteria included a history of congenital heart disease, valvular heart disease, left ventricular systolic dysfunction, cardiomyopathy, previous cardiac surgery, thyroid disease, recent infection, autoimmune or inflammatory diseases, and malignant tumors with a life expectancy shorter than 1 year or those suffering from other end-stage diseases.

### Data Collection

The study collected comprehensive patient data, encompassing age, gender, hypertension, atherosclerotic cardiovascular disease, diabetes, BMI, and other relevant indicators. Simultaneously, the patients’ clinical examination and laboratory findings were gathered, encompassing lymphocyte count, monocyte count, neutrophil count, platelet levels, alanine aminotransferase (ALT), aspartate aminotransferase (AST), total cholesterol (TC), triglycerides (TG), low-density lipoprotein cholesterol (LDL-C), high-density lipoprotein cholesterol (HDL-C), glucose levels (GLU), glycated hemoglobin levels (HbA1c), serum uric acid concentration, and serum creatinine level. Based on the peripheral blood cell counts, we calculated three systemic inflammation markers: SII, SIRI, and AISI. The calculation of SII was performed by multiplying the platelet count with the neutrophil count and dividing it by the lymphocyte count. Similarly, SIRI was determined by multiplying the neutrophil count with the monocyte count and dividing it by the lymphocyte count. Lastly, AISI was computed as a product of neutrophil count, platelet count, monocyte count divided by lymphocyte count.

### Statistical analysis

All the analyses were performed with R and SPSS software. Continuous variables were summarized using the median and interquartile range, while categorical variables were presented as frequencies and percentages. Statistical comparisons between the two groups were performed using χ2 tests for categorical variables, one-way ANOVA tests for normally distributed data, or Kruskal-Wallis tests for non-normally distributed data. The three systemic inflammation markers were analyzed as continuous independent variables, with each variable scaled per 1-unit increment in log-transformed form or divided into tertiles, to explore their associations with the prevalence of PAF. The odds ratios (ORs) and their corresponding 95% confidence intervals (CIs) were estimated using multivariate logistic regression models, incorporating various adjustments. To explore the potential non-linear association between the three systemic inflammation markers and PAF, restricted cubic splines (RCS) analysis was employed. In cases where the RCS analysis revealed a U-shaped, Inverted U-shaped, or L-shaped curve with an identifiable inflection point, we partitioned the data into two distinct segments based on this inflection point and conducted segmented regression analysis separately for each group. Furthermore, the receiver operating characteristic (ROC) curve analysis was employed to determine the optimal cut-off levels of the three systemic inflammation markers for predicting the occurrence of PAF. A value of *p* < 0.05 (two-sided) was considered statistically significant.

## Results

The demographic characteristics of the study cohort are presented in Table 1, comprising a total of 188 individuals included in the analysis. It is worth noting that the PAF group consisted of 91 participants, while the non-PAF group encompassed 97 participants. In general, the baseline characteristics between the non-PAF and PAF groups exhibited significant differences, with the exception of age and HDL-C levels. The levels of SII, SIRI, and AISI exhibited significant elevation in patients with PAF compared to those without PAF. The non-normally distributed continuous variables were subjected to logarithmic transformations for the purpose of analysis. The findings revealed that the significant differences in log-transformed SII, SIRI, and AISI persisted.

**Table 1:** Baseline characteristics of participants included in this study

	Total (*N* = 188)	Non-AF (*N* = 97)	AF (*N* = 91)	*P*
Ages(years)	66(59,72)	63(56,70)	70(64,72)	<0.001
Gender				0.110
Male(%)	92(48.94)	42 (43.30)	50 (54.94)	
Female(%)	96(51.06)	55 (56.70)	41 (45.06)	
Hypertension	125(66.49)	62 (63.91)	63(69.23)	0.441
Diabetes	44(23.40)	25(25.77)	19(20.88)	0.428
Atherosclerotic cardiovascular disease	52(27.66)	24(24.74)	28(30.77)	0.356
BMI(kg/m^2^ )	23.88(21.22,26.57)	23.94(21.24,26.75)	23.65(21.17,26.47)	0.916
Lymphocyte number	1.54(1.23,1.98)	1.74(1.33,2.05)	1.42(1.14,1.83)	0.04
Monocyte number	0.43(0.33,0.53)	0.39(0.32,0.50)	0.47(0.36,0.57)	0.016
Neutrophils number	3.56(2.80,4.43)	3.42(2.74,3.98)	3.88(2.89,4.78)	0.009
Platelet count	208.00(170.25,245.00)	219.00(180.00,251.50)	195.00(162.00,238.00)	0.038
ALT	22.00(17.00,32.43)	21.50(16.45,32.10)	24.00(17.00,32.70)	0.323
AST	28.00(22.00,34.00)	28.00(21.00,32.93)	29.00(22.00,36.00)	0.248
TC(mmol/L)	4.17(3.53,4.72)	4.28(3.64,4.97)	4.09(3.46,4.63)	0.135
TG (mmol/L)	1.22(0.90,1.75)	1.30 (0.93, 1.76)	1.12 (0.89, 1.66)	0.307
LDLC (mmol/L)	2.22(171,2.67)	2.26 (1.79, 2.83)	2.16 (1.62, 2.51)	0.112
HDLC (mmol/L)	1.21(1.02,1.50)	1.26 (1.11, 1.55)	1.15 (0.96, 1.45)	0.016
GLU	5.34(4.69,6.22)	5.21(4.51,6.19)	5.49(4.89,6.24)	0.069
HbA1c	5.90(5.40,6.40)	5.90 (5.70, 6.40)	5.80 (5.30, 6.40)	0.091
Serum uric acid (mmol/L)	329.00(275.25,392.00)	326.00(257.00,382.00)	337.00(283.00,407.00)	0.158
Serum creatinine (mmol/L)	71.39(63.06,84.65)	71.12(64.10,86.00)	72.00(62.72,83.00)	0.780
SII	439.02(339.26,681.84)	398.00 (319.00, 574.00)	518.00 (359.00, 753.00)	0.010
SIRI	0.92(0.64,1.48)	0.78 (0.59, 1.15)	1.13 (0.74, 1.82)	<0.001
AISI	181.10(130.23,319.30)	171.00 (116.00, 254.00)	200.00 (139.00, 400.00)	0.008
LogSII	2.64(2.53,2.83)	2.60 (2.50, 2.76)	2.71 (2.55, 2.88)	0.010
LogSIRI	−0.04(−0.19,0.17)	-0.11 (-0.23, 0.06)	0.05 (-0.13, 0.26)	<0.001
LogAISI	2.26(2.11,2.50)	2.23 (2.06, 2.40)	2.30 (2.14, 2.60)	0.008

ALT alanine transaminase, AST aspartate transaminase, TC total cholesterol, TG triglyceride, LDL-C low density lipoprotein cholesterol, HDL-C high density lipoprotein cholesterol, GLU glucose, HbA1c glycated hemoglobin, SII systemic immune inflammation index, SIRI system inflammation response index, AISI aggregate index of systemic inflammation

### Systemic inflammation markers and PAF proportion

**Fig. 1:**
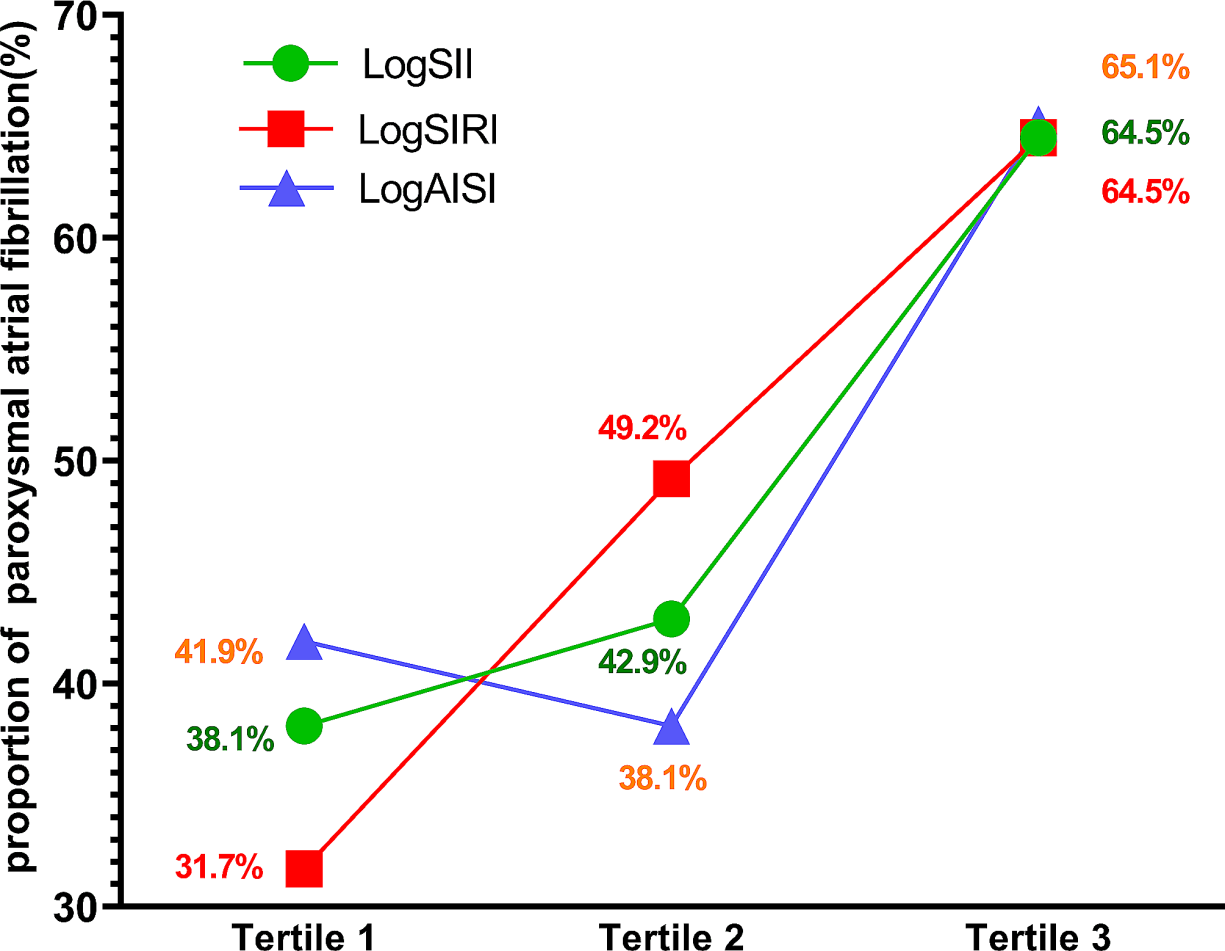
Distribution of PAF proportions among different tertiles of the three systemic inflammation markers

To investigate the association between systemic inflammation markers and the proportion of PAF, we conducted further analyses by dividing subjects into three tertiles based on their log-transformed levels (Refer to Supplementary Table 1 for a detailed breakdown of the grouping). Our study investigated the proportion of PAF across tertiles of logSII, logSIRI and logAISI. For logSII, the number of patients with PAF in tertiles 1–3 was 24, 27 and 40, respectively. Proportion increased from tertile 1 to tertile 3 (38.1%, 42.9% and 64.5%, respectively). In logSIRI, the number of patients with PAF in tertiles 1–3 were 20, 31 and 40, respectively, with proportion of 31.7%, 49.2% and 64.5%. Similarly, in logAISI, the number of patients with PAF in tertiles 1–3 were 26, 24, and 41, respectively, with proportion of 41.9%, 38.1% and 65.1%. Overall, these results demonstrate a gradual escalation in the proportion of PAF as logSII, logSIRI, and logAISI tertiles increase (Fig. 1).

### Systemic inflammation markers and PAF risk

**Fig. 2:**
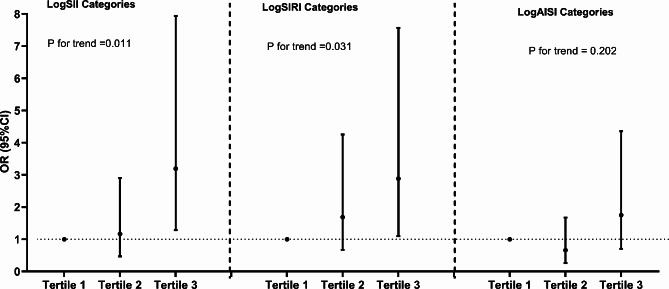
Multivariate-adjusted OR (95% CI) of the relationships between the three systemic inflammation markers and PAF. Adjusted for gender, age, diabetes, hypertension, atherosclerotic cardiovascular disease, body mass index, alanine transaminase, aspartate transaminase, total cholesterol, triglyceride, low density lipoprotein cholesterol, high density lipoprotein cholesterol, glucose, glycated hemoglobin, serum uric acid, serum creatinine

The results of the multivariate logistic regression analysis demonstrate a significant association between elevated tertiles of three systemic inflammation markers and an increased risk of PAF (Table 2). These associations of logSII and logSIRI are significant in both the unadjusted model and the partially adjusted model. From the fully adjusted model, compared to those in the lowest tertile, individuals in the highest logSII, and logSIRI tertiles exhibited a 3.196-fold and 2.884-fold increased risks of PAF, respectively. Conversely, we observed no significant correlation between logAISI and PAF risk among subjects in the highest tertile of logAISI (Fig. 2).

**Table 2:** Logistic regression analysis on predictors of paroxysmal atrial fibrillation

	Model 1		Model 2		Model 3	
	OR(95%CI)	*P*	OR(95%CI)	*P*	OR(95%CI)	*P*
LogSII categories						
Tertile 1	Reference		Reference		Reference	
Tertile 2	1.219(0.598,2.485)	0.586	0.963(0.451,2.055)	0.922	1.166(0.468,2.904)	0.742
Tertile 3	2.955(1.427,6.115)	0.004	2.470(1.137,5.363)	0.022	3.196(1.287,7.937)	0.012
P for trend	0.004		0.023		0.011	
LogSIRI categories						
Tertile 1	Reference		Reference		Reference	
Tertile 2	2.083(1.009,4.300)	0.047	1.620(0.750,3.497)	0.219	1.687(0.669,4.255)	0.268
Tertile 3	3.909(1.859,8.218)	< 0.001	2.818(1.254,6.332)	0.012	2.884(1.099,7.568)	0.031
P for trend	< 0.001		0.012		0.031	
LogAISI categories						
Tertile 1	Reference		Reference		Reference	
Tertile 2	0.852(0.416,1.744)	0.661	0.721(0.338,1.537)	0.397	0.660(0.260,1.674)	0.382
Tertile 3	2.580(1.252,5.317)	0.010	1.994(0.916,4.342)	0.082	1.750(0.703,4.360)	0.229
P for trend	0.01		0.078		0.202	

OR Odds Ratio, CI Confidence Interval

Model 1 was not adjusted for any confounders

Model 2 was adjusted for gender, age, diabetes, hypertension and atherosclerotic cardiovascular disease

Model 3 was adjusted for gender, age, diabetes, hypertension, atherosclerotic cardiovascular disease, body mass index, alanine transaminase, aspartate transaminase, total cholesterol, triglyceride, low density lipoprotein cholesterol, high density lipoprotein cholesterol, glucose, glycated hemoglobin, serum uric acid, serum creatinine

**Fig. 3:**
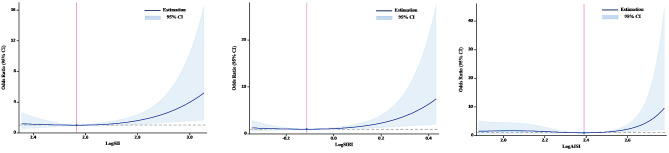
Association between systemic inflammation markers and PAF with the RCS function. The Y-axis shows the odds ratio of having PAF for any value of logSII, logSIRI and logAISI compared to individuals with 2.57 of logSII, -0.11 of logSIRI and 2.39 of logAISI, respectively

The RCS analysis found a U-shaped relationship between logSII, logSIRI, and logAISI and PAF after adjusting for various factors. The inflection point was identified at logSII = 2.57, logSIRI=-0.11, and logAISI = 2.39, respectively (Fig. 3). By utilizing the inflection point, the data was stratified into two distinct groups. Subsequently, segmented regression analysis was conducted on each group separately. The association between logSII, logSIRI, and logAISI with the risk of PAF is statistically significant when logSII ≥ 2.57, logSIRI ≥ -0.11, and logAISI ≥ 2.39 (per standard deviation increase). Specifically, a one standard deviation increase in logSII is associated with a 56% higher risk of PAF (OR = 1.56;95%CI,1.07–2.34), a one standard deviation increase in logSIRI is associated with a 95% higher risk of PAF (OR = 1.95;95%CI,1.24–3.15), and a one standard deviation increase in logAISI is associated with a 150% higher risk of PAF (OR = 2.50;95%CI,1.35–5.25). The results of two piecewise linear regression models are demonstrated in Table 3.

**Table 3:** Effect of standardized systemic inflammation markers on atrial fibrillation: adjusted odds ratios from segmented logistic regression analysis

	OR per SD	95% CI	*P*-value
LogSII (< 2.57)	1.09	0.67, 1.81	0.74
LogSII (≥ 2.57)	1.56	1.07, 2.34	0.026
LogSIRI (< -0.11)	1.31	0.80, 2.26	0.30
LogSIRI (≥ -0.11)	1.95	1.27, 3.15	0.004
LogAISI (< 2.39)	0.98	0.68, 1.43	0.92
LogAISI (≥ 2.39)	2.50	1.35, 5.25	0.008

OR Odds Ratio, CI Confidence Interval

ORs were adjusted for gender, age, diabetes, hypertension, atherosclerotic cardiovascular disease, body mass index, alanine transaminase, aspartate transaminase, total cholesterol, triglyceride, low density lipoprotein cholesterol, high density lipoprotein cholesterol, glucose, glycated hemoglobin, serum uric acid, serum creatinine

### Value of logSII, logSIRI and logAISI in predicting PAF by ROC

**Fig. 4:**
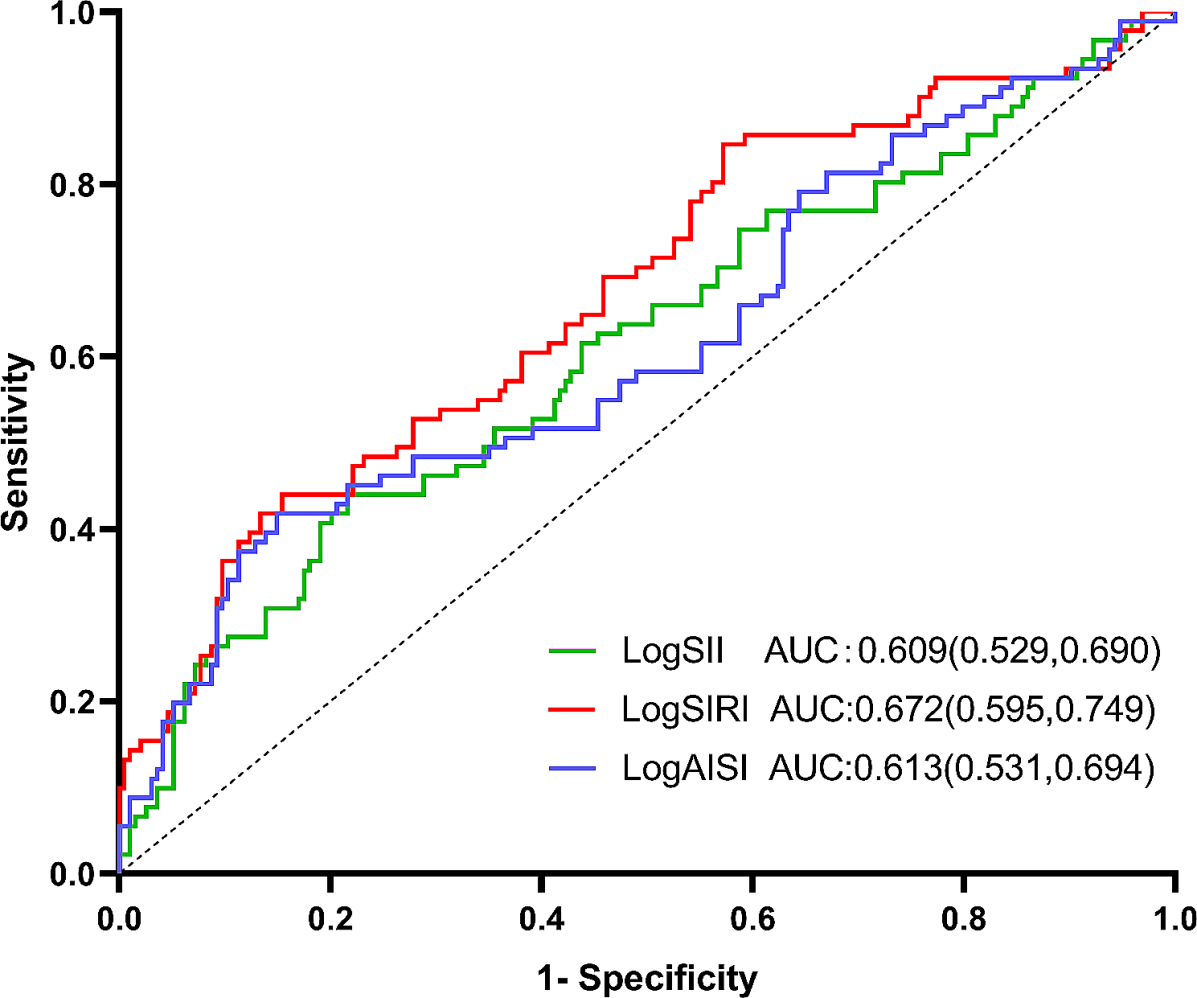
Receiver operating characteristic curves for systemic inflammation markers as a predictor of PAF

We calculated ROC curves to illustrate the performance of logSII, logSIRI, and logAISI to discriminate PAF from non-PAF in all participants. ROC curve analysis of logSII, logSIRI and logAISI showed that they had AUC of 0.609, 0.672 and 0.613.The ability of logSII, logSIRI, and logAISI to predict PAF as shown in Fig. 4. We calculated the cutoff point as 2.710 for logSII, 0.049 for logSIRI and 2.300 for logAISI to estimate the presence of PAF with a sensitivity of 51.6%, 52.7% and 50.5% as well as a specificity of 64.9%, 72.2% and 61.9%, respectively.

## Discussion

In this study, we performed a comprehensive evaluation of different systemic inflammation markers in association with the risk of PAF. The main discoveries of our investigation were as follows: (1) The levels of SII, SIRI and AISI were significantly elevated in PAF patients compared to those without PAF. Furthermore, the proportion of PAF gradually increased with increasing logSII, logSIRI, and logAISI tertiles. (2) Logistic regression analysis indicated that elevated levels of three systemic inflammation markers are associated with an increased risk of PAF. (3) The RCS analysis revealed that when logSII is ≥ 2.57, logSIRI is ≥-0.11, and logAISI is ≥ 2.39, PAF risk increases with the elevation of these systemic inflammation markers in a non-linear relationship. (4) The ROC curve analysis demonstrated favorable sensitivity and specificity of these systemic inflammation markers in detecting the presence of PAF.

This study represents the first attempt to assess the correlation between SII, SIRI, and AISI with PAF. One of the key findings of our study is consistent with previous research conducted by Lin et al., indicating that both SII and SIRI play a significant role in predicting AF [12]. In contrast to these studies, the objective of our study was to investigate the correlation between PAF, a subtype of AF, and systemic inflammatory markers. This suggests that elevated levels of these markers are strongly associated with an increased likelihood of PAF development. In addition, we have additionally employed RCS analysis to explore the non-linear correlation between three systemic inflammation markers and PAF. It is noteworthy that Lin et al. did not perform ROC curve analysis to determine the optimal cut-off levels of systemic inflammation markers for predicting AF occurrence. Another significant finding from our study was that logSIRI may be the optimal systemic inflammation marker for estimating the risk of PAF, as it yields the most significant ORs (95% CIs) and performs well in segmented regression analysis and ROC analyses. Our study demonstrates that the assessment of SII, SIRI, and AISI levels can serve as valuable tools in identifying high-risk patients for PAF, facilitating targeted monitoring and early intervention strategies. Regular surveillance of these biomarkers in individuals at risk can significantly contribute to the detection and management of PAF, potentially impeding its progression. Considering the well-established association between inflammation and PAF, exploring anti-inflammatory therapies may prove advantageous for patients exhibiting elevated SII and SIRI levels.

The exceptional performance of SII, SIRI, and AISI as emerging biomarkers has been demonstrated across a diverse spectrum of diseases, including cancers and cardiovascular diseases [13, 16–18]. In comparison to conventional markers of inflammation, the three systemic inflammation markers exhibit more favorable characteristics as indicators of inflammatory status and have consistently demonstrated superior predictive power and prognostic value across multiple studies [19–22]. According to Yang’s research, the predictive capacity of SII surpasses that of traditional risk factors for major cardiovascular events in patients with coronary artery disease (CAD) who have undergone coronary intervention [17]. Consistently, another study has reported a robust association between SII and SIRI with both cardiovascular and all-cause mortality, underscoring the imperative of addressing systemic inflammation for enhanced preventive strategies [23]. Also, the risk of major adverse cardiovascular events (MACE) in patients with acute coronary syndrome undergoing percutaneous coronary intervention was significantly elevated with increasing tertiles of AISI and SIRI [13].

AF is a prevalent cardiac arrhythmia that significantly increases the risk of stroke and mortality. Inflammation, which plays a crucial role in both the initiation and maintenance of AF, represents an important therapeutic target for intervention. Despite numerous studies demonstrating the involvement of inflammatory factors and their resultant products, such as IL-1β, IL-6, IL-8,IFN-γ, TNF-α and CRP in the pathogenesis of AF [8, 24–27], there remains a lack of research investigating the association between these novel systemic inflammatory markers and AF or PAF. The association between a higher neutrophil-to-lymphocyte ratio (NLR) and the onset of AF, as well as its recurrence following AF ablation, is significantly strengthened [28].The pre-ablation NLR emerged as a robust and independent predictor of AF recurrence following cryoablation [29, 30].Moreover, based on Kaplan’s research findings, an elevated SII level functions as an independent prognostic indicator for the recurrence of AF [30]. Additionally, a study has demonstrated that SII can serve as an independent predictor for the development of new-onset atrial fibrillation (NOAF) following STEMI [31]. Although the lymphocyte, neutrophil, and platelet counts were significantly lower in patients with PAF compared to those without PAF in our study, it is noteworthy that PAF patients exhibited elevated levels of systemic immune-inflammation indices (SII, SIRI, and SIAI), indicating the presence of a systemic inflammatory process associated with PAF. Leukocyte activation and inflammation play a crucial role in the development of atrial fibrillation, leading to electrical and structural remodeling [26, 32]. Inflammatory markers can be used to assess the risk of atrial fibrillation in individuals with varying levels of inflammation. Therefore, these novel systemic inflammatory markers, in conjunction with conventional inflammation factors, may offer a straightforward and dependable approach to evaluate the risk of PAF among individuals with varying levels of inflammatory status.

Several limitations associated with the present study warrant mention. First, this study was a retrospective analysis based on prospectively collected data, and all enrolled patients were from a single centre. The presence of imbalances in baseline characteristics is likely to occur in small single-center studies like this, which may contribute to the associations being influenced by significant differences in baseline characteristics. Second, the inclusion of specific inflammatory biomarkers was not considered in this study, and the evaluation was limited to only three closely related cell-based parameters, which represents a constraint in terms of assessing inflammatory indicators. Third, the sample size in this study was relatively small, and data splitting and cross-validation were not be applied, which may lead to overfitting. What is more, the observational design of the study identifies only an association and not causality. Thus, further studies with larger patient populations will be needed to validate our findings.

## Conclusion

In conclusion, the proportion of PAF gradually increased with increasing logSII, logSIRI, and logAISI tertiles. Compared to those in the lowest tertiles, the PAF risks in the highest logSII and logSIRI tertiles were 3.2-fold and 2.9-fold, respectively. The RCS analysis revealed a non-linear relationship between the elevation of systemic inflammation markers and PAF risk. The ROC curve analysis of logSII, logSIRI, and logAISI yielded AUC values of 0.6, 0.7, and 0.6, respectively. Moreover, these systemic inflammation markers exhibited favorable sensitivity and specificity in detecting the presence of PAF.

### Electronic supplementary material

Below is the link to the electronic supplementary material.

Supplementary Material 1

## Data Availability

Data is provided within the manuscript or supplementary information files. The datasets used and/or analyzed during the current study are available from the corresponding author on reasonable request.
