# Supplementary material for: The association between systemic inflammation markers and paroxysmal atrial fibrillation
Source: BMC Cardiovasc Disord. 2024 Jul 4;24:334. doi: 10.1186/s12872-024-04004-9 (PMC11223271; doi:10.1186/s12872-024-04004-9)
Supplement: Supplementary file 1 — Supplementary Material 1 [file 12872_2024_4004_MOESM1_ESM.docx]

| Supplementary Table 1 Systemic inflammation markers and PAF prevalence | | | | | |
| --- | --- | --- | --- | --- | --- |
|  |  | Non-PAF  (n) | PAF  (n) | PAF  Prevalence(%) | P |
| LogSII |  |  |  |  | 0.007 |
|  | Tertile1（≤2.56） | 39 | 24 | 38.1 |  |
|  | Tertile2（2.57-2.76） | 36 | 27 | 42.9 |  |
|  | Tertile3（≥2.77） | 22 | 40 | 64.5 |  |
| LogSIRI |  |  |  |  | 0.001 |
|  | Tertile1（≤-0.14） | 43 | 20 | 31.7 |  |
|  | Tertile2（-0.13-0.09） | 32 | 31 | 49.2 |  |
|  | Tertile3（≥0.10） | 22 | 40 | 64.5 |  |
| LogAISI |  |  |  |  | 0.005 |
|  | Tertile1（≤2.17） | 36 | 26 | 41.9 |  |
|  | Tertile2（2.18-2.43） | 39 | 24 | 38.1 |  |
|  | Tertile3（≥2.44） | 22 | 44 | 65.1 |  |
